# Development of a Conserved Chimeric Vaccine for Induction of Strong Immune Response against *Staphylococcus aureus* Using Immunoinformatics Approaches

**DOI:** 10.3390/vaccines9091038

**Published:** 2021-09-18

**Authors:** Rahul Chatterjee, Panchanan Sahoo, Soumya Ranjan Mahapatra, Jyotirmayee Dey, Mrinmoy Ghosh, Gajraj Singh Kushwaha, Namrata Misra, Mrutyunjay Suar, Vishakha Raina, Young-Ok Son

**Affiliations:** 1 Kalinga Institute of Industrial Technology (KIIT), School of Biotechnology, Deemed to Be University, Bhubaneswar 751024, India; rahulksbt@gmail.com (R.C.); soumyaranjanmahapatra685@gmail.com (S.R.M.); jmdey1997@gmail.com (J.D.); namrata@kiitincubator.in (N.M.); mrutyunjay@kiitincubator.in (M.S.); 2 Kalinga Institute of Medical Sciences, Kalinga Institute of Industrial Technology (KIIT), Deemed to Be University, Bhubaneswar 751024, India; panchanan.sahoo@kims.ac.in; 3 KIIT-Technology Business Incubator (KIIT-TBI), Kalinga Institute of Industrial Technology (KIIT), Deemed to Be University, Bhubaneswar 751024, India; mringhs2010@gmail.com (M.G.); gajraj@kiitincubator.in (G.S.K.); 4 International Centre for Genetic Engineering and Biotechnology (ICGEB), Transcription Regulation Group, New Delhi 110067, India; 5 Department of Animal Biotechnology, Faculty of Biotechnology, Jeju National University, Jeju 63243, Korea; 6 Bio-Health Materials Core-Facility Center, Jeju National University, Jeju 63243, Korea; 7 Practical Translational Research Center, Jeju National University, Jeju 63243, Korea; 8 Interdisciplinary Graduate Program in Advanced Convergence Technology and Science, Jeju National University, Jeju 63243, Korea

**Keywords:** *Staphylococcus aureus*, SdrD, SdrE, immunoinformatics, multi-epitope vaccine

## Abstract

*Staphylococcus aureus* is one of the most notorious Gram-positive bacteria with a very high mortality rate. The WHO has listed *S. aureus* as one of the ESKAPE pathogens requiring urgent research and development efforts to fight against it. Yet there is a major layback in the advancement of effective vaccines against this multidrug-resistant pathogen. SdrD and SdrE proteins are attractive immunogen candidates as they are conserved among all the strains and contribute specifically to bacterial adherence to the host cells. Furthermore, these proteins are predicted to be highly antigenic and essential for pathogen survival. Therefore, in this study, using the immunoinformatics approach, a novel vaccine candidate was constructed using highly immunogenic conserved T-cell and B-cell epitopes along with specific linkers, adjuvants, and consequently modeled for docking with human Toll-like receptor 2. Additionally, physicochemical properties, secondary structure, disulphide engineering, and population coverage analysis were also analyzed for the vaccine. The constructed vaccine showed good results of worldwide population coverage and a promising immune response. For evaluation of the stability of the vaccine-TLR-2 docked complex, a molecular dynamics simulation was performed. The constructed vaccine was subjected to in silico immune simulations by C-ImmSim and Immune simulation significantly provided high levels of immunoglobulins, T-helper cells, T-cytotoxic cells, and INF-γ. Lastly, upon cloning, the vaccine protein was reverse transcribed into a DNA sequence and cloned into a pET28a (+) vector to ensure translational potency and microbial expression. The overall results of the study showed that the designed novel chimeric vaccine can simultaneously elicit humoral and cell-mediated immune responses and is a reliable construct for subsequent in vivo and in vitro studies against the pathogen.

## 1. Introduction

*Staphylococcus aureus* is one of the WHO-declared ESKAPE pathogens for causing community- and healthcare-associated infections [1]. *S. aureus* expresses an array of virulence factors including cell wall adhered proteins to colonize host tissues by specifically binding to different host matrix substrates, such as fibronectin, fibrinogen, collagen, and cytokeratin [2,3,4]. These proteins particularly include sdrD and sdrE, which have N-terminal secretory signal peptide, followed by an A domain, B repeat regions, R domain-containing serine–aspartate repeats, membrane-spanning region, and a charged cytoplasmic tail.

Additionally, these nosocomial bacteria were shown to possess the ability to form biofilms on indwelling medical devices, including implanted artificial heart valves, catheters, and joint prosthetics [5]. The emergence and dissemination of multidrug-resistant strains are the main reasons determining the challenge in dealing with these infections [6]. The development of resistance to many antibiotics by *S. aureus* has involved the acquisition of determinants by horizontal gene transfer of mobile genetic elements [7]. Despite several attempts to develop experimental *S. aureus* vaccines and immunotherapeutics, none has proven successful in preventing staphylococcal infections in humans due to multiple challenges, particularly, the rapid development of multi-drug-resistant strains and extensive clinical trials regimen a variety of potential virulence factors produced by this organism [8]. Several possibilities were studied in the past, including capsular polysaccharides of types 5 and 8, IsdB, an iron scavenging protein, and passive immunization against clumping factor A and lipoteichoic acid. Regardless of the fact that these experiments showed great promise in mouse models, but they failed in clinical trials [9,10]. Similarly, Veronate, an immunoglobulin preparation for invasive *S. aureus* illness produced from a pool of high titer anti-ClfA serum samples, showed promise in an early study but failed in phase III studies [11]. A monoclonal anticlumping factor was also added. Tefibazumab (Aurexis) (https://en.wikipedia.org/wiki/Tefibazumab, accessed on 17 Septermber 2021) is an antibody that was shown to be effective against an *S. aureus* bacterial load [12]. Panton–Valentine leukocidin [13], a-hemolysin, and a vaccine comprising IsdA, IsdB, SdrD, and SdrE [14] are among the protein antigens being studied in early clinical trials by Pfizer, Novartis, Novadigm, and GSK [15]. Pier et al. have clearly mentioned the *S. aureus* vaccines. Vaccines against CP antigens, immunity to ClfA and vaccination against IsdB have all failed in human trials [16]. Against this backdrop, it was suggested for a multi-antigen strategy y appropriate combination of antigens and underpinning epitopes that can elicit both humoral and cellular immune responses [15,17].

**Figure 1:**
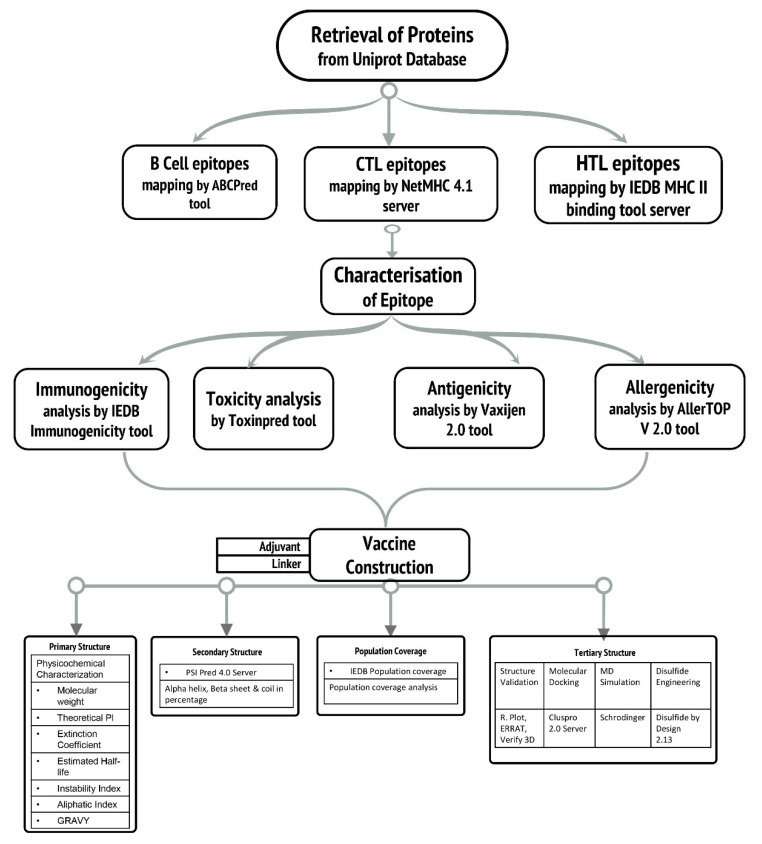
Schematic representation of the workflow for the development of a multi-epitope vaccine against *S. aureus* infections.

Hence, the objective of the study was to perform in silico prediction of B and T epitopes from antigenic protein targets *viz.*, SdrD and SdrE in order to design a subunit vaccine against Staphylococcal infections. Furthermore, to improve vaccine efficacy, the final multi-epitope vaccine construct was designed by assembling the best epitopes, Phenol soluble modulin α4 as adjuvant and appropriate linkers. Subsequently, the physicochemical properties and the secondary and tertiary structures of the vaccine were predicted. Furthermore, the interaction analysis of the vaccine construct with the immune receptor (TLR-2) was evaluated by docking and molecular dynamics simulations, revealing high affinity and complex stability. Codon adaptation and in silico cloning revealed higher expression of designed subunit vaccine in *E. coli* expression system. The immune simulation was performed to confirm the immunogenic potential of the designed vaccine construct. The results obtained from various in silico experiments indicate the potency of the predicted vaccine candidate as a probable therapeutic against *S. aureus*. Figure 1 shows the overall computational workflow used in this study.

## 2. Methodology

### 2.1. Retrieval of Protein Sequences

The protein sequences of sdrD (O86488) and sdrE (O86489) of *S. aureus* were retrieved from the UniProt database (http://www.uniprot.org/uniprot, accessed on 17 September 2021) for further analysis. The protein sequences are presented in Table S1.

### 2.2. Epitopic Region Prediction of B-Cell

B-cells provide humoral immunity by secreting immunoglobulins which can neutralise antigen upon binding. B-cell epitopes are two types, linear (continuous) and conformational (discontinuous) [18]. In the case of vaccine design, however, only the linear epitopes are considered. Linear/continuous epitope comprises a single continuous stretch of amino acids within a protein sequence that can react with anti-protein antibodies. Unlike discontinuous epitopes, the linear epitopes are easy to design, as they do not require tertiary structure information. Linear peptides were also reported to significantly evoke neutralising antibodies against the pathogen [19]. Linear B-cell epitopic regions were primarily recognized using the ABCpred server (https://webs.iiitd.edu.in/raghava/abcpred/ABC_submission.html, accessed on 17 September 2021). ABCPred is a consistent algorithms-based webserver specifically used for the appropriate prediction of linear B-cell epitopes. As the B-cell epitope is present on the cell surface, exomembrane topology is considered as one of the essential parameters. In ABCpred, the length of epitopes 10-mer, the threshold of 0.51%, and default specificity of 75% was selected.

### 2.3. Prediction of Cytotoxic T-Lymphocyte (CTL) and Helper T-Lymphocyte (HTL) Epitopes

An effective immune response depends on the specificity and diversity of the antigen-binding to the human leukocyte antigen (HLA) [20] class I (recognises CD8+ T-cells), and class II (recognises CD4+ T-cells) alleles [21]. The cytotoxic T lymphocyte (CTL) epitopes from the conserved peptides were predicted using the NetMHCpan 4.1 server available at http://www.cbs.dtu.dk/services/C-ImmSim-10.1/ (accessed on 17 September 2021) that is based on the neural network architecture [22]. This predicts candidate epitopes based on the processing of the peptides in vivo which also covers 12 HLA-I supertypes (A1, A2, A3, A24, A26 B7, B8, B27, B39, B44, B58, and B62). The threshold for strong binder and weak binder were, respectively, set at 0.5% and 2% rank. Sorting by prediction score was selected.

Conserved peptides were also tested for predicting epitopes that interact with MHC class II molecules by selecting all the alleles in the IEDB MHC class II binding prediction tool (http://tools.immuneepitope.org/mhcii/, accessed on 17 September 2021) [23]. IEDB predictions use default and consensus calculations for predictions. Twenty-seven human leukocyte antigens (HLA) were evaluated. According to the IEDB server, the lowest consensus scores of the peptides were chosen to be the best binders and a lower percentile rank indicates higher affinity. The selection criterion was a cut-off of IC50 ≤ 50 and percentile rank < 1.

### 2.4. Evaluation of Antigenic, Allergenic, Immunogenicity, and Toxicity

Each of the epitopes was subjected for assessment of antigenicity by VaxiJen 2.0 (http://www.ddg-pharmfac.net/vaxijen/VaxiJen/VaxiJen.html, accessed on 17 September 2021), a server for alignment-independent prediction of protective antigens and which allows antigen classification solely based on the physicochemical properties of proteins [24]. The threshold for the model was set to be 0.4. To analyse the non-allergenic nature, the vaccine sequence was examined using AllerTOP v2.0 (https://www.ddg-pharmfac.net/AllerTOP/, accessed on 17 September 2021) [25]. The amino acid sequences of SDR proteins were entered into the program separately and other parameters remained as defaults. The immunogenicity score of all the predicted MHC I epitopes was checked using the IEDB class I immunogenicity server which is available at http://tools.iedb.org/immunogenicity/ (accessed on 17 September 2021). The linear epitopes were checked for potential toxicity using ToxinPred (http://crdd.osdd.net/raghava/toxinpred/, accessed on 17 September 2021) server [26].

### 2.5. Epitope Selection Criteria

In the process of epitope selection, only peptides that exhibited high antigenicity score, non-allergenic, non-toxicity, binding to the maximum number of HLA alleles were selected for further analysis.

### 2.6. Vaccine Construction

MHC class I and II binding epitopes were selected based on their high binding affinity and non-allergenic nature for vaccine construction. In the next step, adjuvant Phenol soluble modulin α4 (Accession no. A9JX08) protein was selected based on a literature study to develop the effectiveness of the vaccine. The EAAAK linker at the N-terminus of the vaccine construct was used to fuse the adjuvant and further B-cell epitopes get connected with the KK linker. MHC-I epitopes were fused by AAY, whereas MHC-II epitopes were fused by GPGPG linkers. These linkers were shown to be helpful for differentiation and improvement of epitope presentation [27].

### 2.7. Estimation of Population Coverage

The distribution of HLA alleles, as well as their expression, could vary throughout the world according to the difference in regions and ethnicities [28]. As a result, successful vaccine production necessitates a global assessment of HLA allele distribution. In this study, the distribution of HLA alleles for potential CTL and HTL epitopes was evaluated with the IEDB population coverage tool (http://tools.iedb.org/population/, accessed on 17 September 2021) [29]. This server is designed to estimate the population coverage of epitopes from different regions based on the distribution of their MHC-binding alleles. By keeping the default parameters, sixteen geographical areas such as East Asia, Northeast Asia, South Asia, Southeast Asia, Southwest Asia, Europe, East Africa, West Africa, Central Africa, North Africa, South Africa, West Indies, North America, Central America, South America, and, Oceania were selected for population coverage.

### 2.8. Analysis of Solubility and Physicochemical Properties and Secondary Structure

Solubility of the designed vaccine was assessed using SOLpro (https://scratch.proteomics.ics.uci.edu, accessed on 17 September 2021) server. ProtParam server (http://web.expasy.org/protparam/, accessed on 17 September 2021) [30] was employed to calculate several physiochemical properties of the vaccine construct, namely amino acid composition, instability index, theoretical PI, in vitro and in vivo half-life, aliphatic index, and grand average of hydropathy (GRAVY). The instability index predicts whether a protein is stable or unstable. A protein is computed as stable if the predicted instability index is below 40 while a value above 40 denotes unstable protein.

The secondary structure of the multi-epitope vaccine was predicted using PSIPRED v3.3 (http://bioinf.cs.ucl.ac.uk/psipred/, accessed on 17 September 2021) server [31] with default parameters.

### 2.9. Vaccine Construct’s Antigenicity and Allergenicity Profiling

The antigenicity and allergenicity profiles of the vaccine construct were also determined. The VaxiJen server [24] was employed to assess the antigenicity of the vaccine construct, and the AllerTop server [25] was used to evaluate the non-allergenic nature of the vaccine construct.

### 2.10. Prediction of Interferon-Gamma Inducing Epitopes

To design a vaccine with the highest ability to induce the immune system, we identified interferon-gamma inducing epitopes by using the “IFNepitope” server (https://webs.iiitd.edu.in/raghava/ifnepitope/design.php/, accessed on 17 September 2021). This software is based on a dataset comprising of IFN-gamma-inducing and non-inducing MHC class II binders through various approaches that have a maximum accuracy of 81.39% [32]. Our sequences were analyzed by motif and SVM hybrid and IFN-gamma versus non-IFN-gamma.

### 2.11. Three-Dimensional Modelling and Validation

Homology modelling of the final vaccine construct was performed using the Robetta server (https://robetta.bakerlab.org/, accessed on 17 September 2021) [33], applied for visualising 3D structures of proteins. Additionally, with the help of ProSA-web (https://prosa.services.came.sbg.ac.at/prosa.php, accessed on 17 September 2021) [34], the tertiary structure validation was accomplished. Another server used for the validation of the tertiary structure is ERRAT (http://services.mbi.ucla.edu/ERRAT/, accessed on 17 September 2021). We used the ERRAT server in order to analyze the statistics of non-bond interaction between different types of atoms [35]. Additionally, the Ramachandran plot was investigated by using the PROCHECK (https://servicesn.mbi.ucla.edu/PROCHECK/, accessed on 17 September 2021) server [36] to find out the relative proportion of amino acid which falls in the favored region, relative to other regions. Verify 3D (https://saves.mbi.ucla.edu/, accessed on 17 September 2021) was also used to confirm the validation of the developed model.

### 2.12. Disulphide Engineering

Disulphide engineering is a novel approach for creating disulphide bonds into the target protein structure. Disulphide bonds are covalent interactions that help in increasing the protein stability along with the examination of protein interactions and dynamics [37,38]. Therefore, the selected refined vaccine model was subjected to the Disulphide by Design 2.12 [39] web platform to perform disulphide engineering. Initially, the refined protein model was uploaded and run for the residue pair search that can be used for the disulphide engineering purpose. Potential residue pairs were selected for mutation with cysteine residue using create mutate function of the Disulfide by Design 2.12 server (http://cptweb.cpt.wayne.edu/DbD2/index.php, accessed on 17 September 2021).

### 2.13. Molecular Docking Analysis

To determine the peptide–protein docking, the ClusPro server (https://cluspro.bu.edu/login.php, accessed on 17 September 2021) [40] was employed. Toll-like receptors are integral membrane proteins that express on the sentinel cells of innate immunity and generate the antiviral response. The structural coordinates of the TLR 2 (PDB ID: 3A7C) were retrieved from the Protein Data Bank (https://www.rcsb.org/, accessed on 17 September 2021). The TLR 2 was used as receptor molecules for the docking with vaccine protein as ligand using ClusPro 2.0 server (https://cluspro.bu.edu/publications.php, accessed on 17 September 2021) that is based on PIPER, a Fast Fourier Transform (FFT) correlation approach for protein docking with pairwise interaction potential [40]. Each docking output had thirty models which were generated from the three sequential steps including rigid-body docking, clustering of lowest energy structure, and structural refinement. The docked structures were visualised through PyMol (http://www.pymol.org, accessed on 17 September 2021) to analyze the interactions between vaccine and TLR-2.

### 2.14. Dynamics Simulations for Vaccine Stability

Molecular dynamics (MD) simulation is essential to determine the stability of the receptor-ligand complex. The MD simulation of the complex between TLR-2 (as a receptor) and vaccine (as a ligand) was performed using the Schrodinger package. The MD simulation workflow included protein preparation, system building, MD simulation, and trajectory analysis. The whole workflow will be carried out using the Maestro interface of Schrodinger. Protein structures were imported and prepared using Protein Preparation Wizard. It includes hydrogen addition, protonation, and refinement of coordinates by energy minimization using the OPLS3e force field [41]. The simulation system was prepared using the System Builder tool. The protein complex was kept in an orthorhombic box extended 10 Å from the protein complex. The system was solvated using the TIP3P water model [42] with 150 mM NaCl to neutralize counter-charges. The system was minimized and pre-equilibrated using default parameters before long-run MD simulation. Finally, the system was subjected to 100 ns the NPT ensemble with default parameters where temperature (300 K) and pressure (1 atm) were maintained. The simulation trajectory was visualized in Maestro and analysis was carried out using the simulation interaction diagram tool. The data were plotted as root mean square deviation (RMSD), and root mean square fluctuations (RMSF) graph.

### 2.15. Immune Simulation of the Vaccine Construct

To evaluate that the developed vaccine construct elicits a strong immune response in the mouse model, the online dynamic immune simulation C-ImmSim server (https://www.iac.cnr.it/~filippo/c-immsim/index.html, accessed on 17 September 2021) [43] was employed. The advanced C-ImmSim server uses the amino acid strings in place of Bit strings. As per the suggested literature, a dosing interval of 1 month is prescribed for the immunization of the vaccine. Therefore, we selected a one-month interval and identified the dose-dependent immunological response of our vaccine candidate. All of the parameters were left at their default values during the experiment, with the exception of the time steps, which were set to 1, 84, and 168 (time step 1 is injection at time ¼ 0), and the number of simulation steps, which was set to 1050.

### 2.16. In Silico Cloning and Optimisation

To test the vaccine construct’s expressibility in *E. coli* cells, *in-silico* cloning was used. For the codon optimization investigation, the Java Codon Adaptation Tool (JCAT) (http://www.jcat.de/, accessed on 17 September 2021) was utilized to ensure the relationship between codon usage and gene expression. The produced vaccine sequence was employed as input, and the expression host was chosen to be the *E. coli* K12 strain. During the run, the options to avoid rho independent transcription terminators, bacterial ribosome binding sites, and restriction enzyme cleavage sites were chosen. The JCat server output includes percentage GC content and codon adaptation index (CAI), which can be used to ensure high protein expression. The pET-28a (+) vector was chosen for cloning the modified vaccine construct inside *E. coli*, followed by the introduction of the *EcoR*I and *BamH*I restriction sites at the N and C terminal, respectively. Finally, the optimized codon sequence was cloned using the SnapGene restriction cloning tool, and the pET28a (+) plasmid vector was used to insert it.

## 3. Result

### 3.1. Epitopic Region Prediction of B-Cell

The FASTA sequences of SDR protein (SdrD and SdrE) upon peptide mapping by ABCPred yielded a total of 87 epitopes at the default threshold >0.51 and window length of 10 (Table S2). The two peptides (NEENKKVDAK of SdrD and DTGGGDGTVK of SdrE) of 10 mer that had the highest score by ABCpred server were selected for further analysis of antigenicity, allergenicity, and toxicity (Table 1).

### 3.2. Prediction of Cytotoxic T-Lymphocyte (CTL) and Helper T-Lymphocyte (HTL) Epitopes

MHC-I antigenic determinants activate an immune cellular response. This type of response normally activates cytotoxic cells. The *S. aureus* protein sequences were analyzed by NetMHCpan 4.1 server to identify the most immunodominant regions. Peptides with the highest binding affinity scores in each protein were identified as high-potential CTL epitope candidates. The NetMHCpan 4.1 predicted MHC-I epitopes are listed in Table S3 with encountering MHC alleles, average rank scores, conservancy prediction, and allergenicity assessment. Additionally, the final chosen top 20 sequences of epitopes for vaccine construction are listed in Table 2.

HTL epitopes for all structural proteins were predicted using the IEDB server for human MHC-II alleles. The epitopes with percentile rank ≤ 1 and SMM align IC50 score below 50 were selected for further analysis (Table S4). Among these, the top two epitopes viz., KRLNTRMRIAAVQPS of SdrD and DTEFTIDNKVKKGDT of SdrE based on their least percentile rank and their high affinity with respect to all the HLA supertypes were selected for inclusion in vaccine construct. (Table 3).

### 3.3. Evaluation of Antigenicity, Allergenicity, Toxicity, and Immunogenicity

The vaccine needs to be antigenic and non-allergic in nature and induce humoral as well as cell-mediated immune responses against the targeted pathogen. On further analysis, the epitopes were observed to be antigenic with a high probability score predicted by the VaxiJen v2.0 server. The epitopes were also detected as non-allergen, checked using the AllerTOP v2.0 server. Toxicity predictions using the ToxinPred server indicated that none of the selected epitopes were toxic to humans. The immunogenicity score of the chosen CTL epitopes was predicted by IEDB Class I immunogenicity tool. All the epitopes exhibited high immunogenicity.

### 3.4. Vaccine Construction

**Figure 2:**
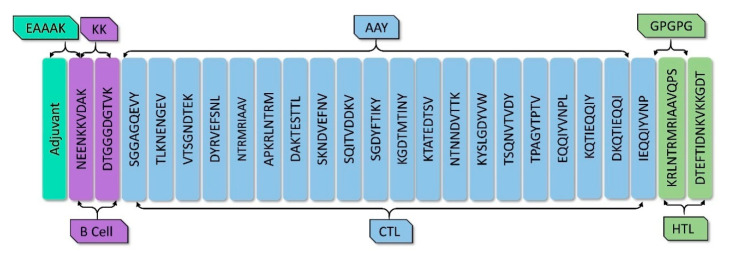
The structural arrangement of B- and T-cell epitopes along with linkers and adjuvant for the final multi-epitope vaccine construct.

The vaccine construct was designed with the inclusion of high scored T-cell and B-cell epitopes predicted from various epitope prediction tools and exhibiting high immunogenicity, non-toxicity, non-allergenic and strong binding affinity to a maximum number of HLA alleles [44]. The two B-cell epitopes (NEENKKVDAK of SdrD and DTGGGDGTVK of SdrE) were linked by the KK linker. For the CTL structure, ten epitopes from sdrD and ten epitopes from SdrE were selected. The CTL epitopes were linked by the AAY linker, and finally, for Helper T-cell (HTL) structure, one epitope from each protein (KRLNTRMRIAAVQPS of SdrD and DTEFTIDNKVKKGDT of SdrE) was selected. The HTL epitopes were linked by the GPGPG linker. Phenol soluble modulin α4 (UniProt Id: A9JX08), TLR-2 agonist, was added with EAAAK linker to the N- terminal end as an adjuvant to enhance the immunogenic property of the vaccine construct (Figure 2). The final epitopes selected for the vaccine construct are mentioned in Table S5.

### 3.5. Estimation of Population Coverage

Since different MHC-I and MHC-II HLA alleles are exposed at a different frequency (population/individual) in different ethnicities across the world, hence the analysis of how many individuals will be covered by the respective HLA alleles of the predicted epitopes is a vital part of designing an effective vaccine. The population coverage analysis results of the designed vaccine construct showed approximately 98.95% coverage across worldwide, particularly highest in Europe (99.66%), East Africa (99.49%), West Africa (99.50%), Central Africa (99.61%), North America (99.19%) (Table S6).

### 3.6. Analysis of Solubility and Physicochemical Properties and Secondary Structure

The multi-epitope vaccine was predicted as soluble with probability 0.536149 by the SOLpro server of the Scratch protein predictor tool. The physicochemical properties of the final vaccine construct including molecular weight, half-life, instability index, net charge, hydrophobicity, GRAVY, and aliphatic index are presented in Table S7. Expasy’sProtParam tool classifies the vaccine as stable with an instability index of value 24.73. The molecular weight of the construct was found to be 35.60 kDa which is ideal as a small size construct is easy to handle and purify during experimental evaluation. Aliphatic index and GRAVY were noted of value 70.03 and −0.438 reflecting high thermostability and hydrophilic nature, respectively. The theoretical p*I* (5.79) results showed that the vaccine was naturally acidic. The total number of positively charged residues (Arg+Lys) and negatively charged residues (Asp+Glu) were 36 and 35, respectively. The estimated half-life in mammals, yeast, and *E. coli* was observed to be 30 h, >20 h, and >10 h, respectively.

**Figure 3:**
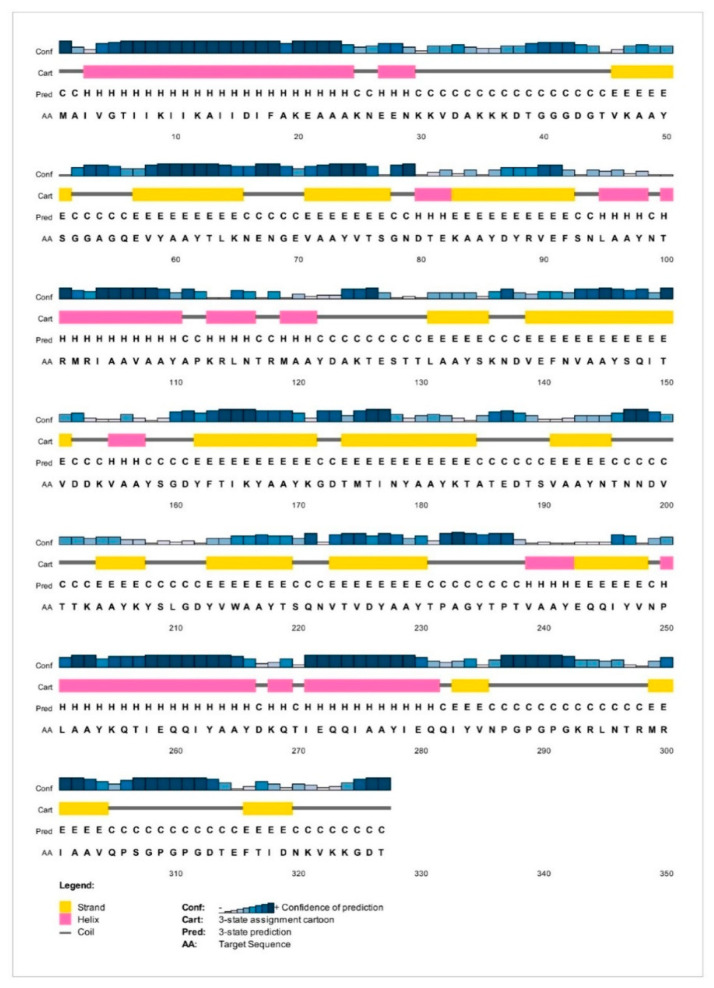
Secondary structure prediction of the final multi—epitope vaccine constructs by using the PSIPRED tool.

Based on PSIPRED server results, the protein vaccine consists of 26.60% alpha-helix (H), 34.86% extended strand (E), and 38.53% random coil (C) secondary structural elements. Schematic representation of the secondary structure prediction results was represented in Figure 3.

### 3.7. Vaccine’s Antigenicity and Allergenicity Profiling

The antigenicity of the whole vaccine construct was observed to be high with a score of 0.9422 as predicted by VaxigenV2.0, suggesting that the vaccine is immunogenic and can trigger a strong immune response. AllerTOPV2.0 tool also classified the construct to be non-allergen against humans.

### 3.8. Prediction of Interferon-Gamma Inducing Epitopes

Using the IFNepitope server, the IFN-gamma inducing epitopes were identified from MHC-II binding epitopes fragments in the final vaccine construct. A total of 112 epitopes were predicted as positive (Table S8). The amino acid residues of the vaccine, which are located in the 231–246 region, showed the highest score.

### 3.9. Three-Dimensional Modelling and Validation

**Figure 4:**
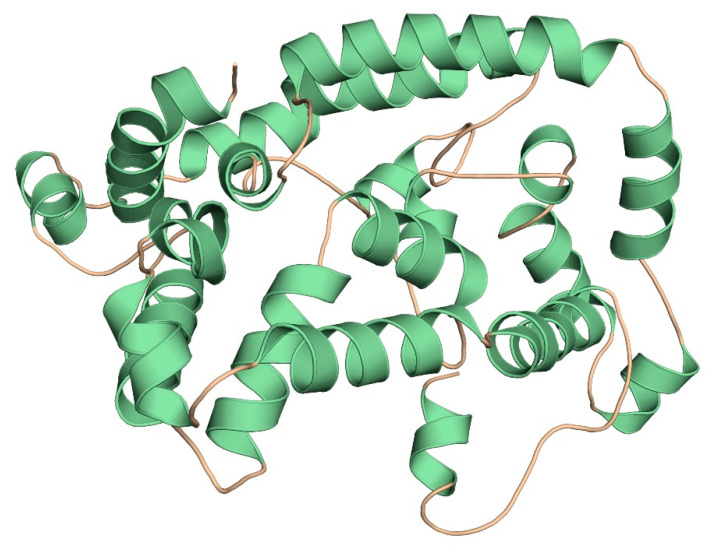
Homology modelling of the three-dimensional structure of the final multi-epitope vaccine construct.

**Figure 5:**
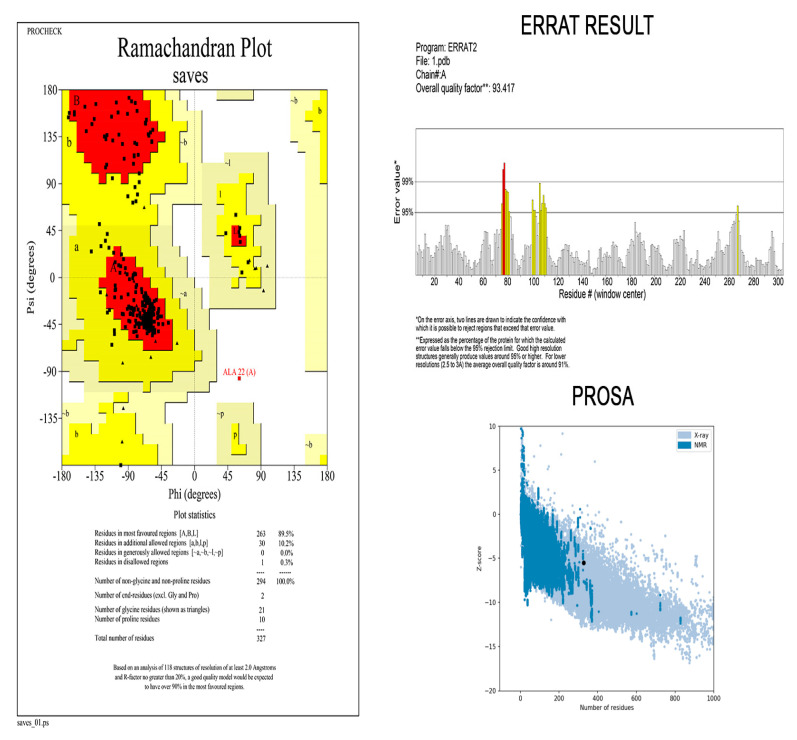
Developed multi—epitope vaccine structure validation results confirmed the model to be reliable and accurate.

The primary 3D model of the multi-epitope vaccine was generated by the Robetta server (Figure 4) and was visualized by Pymol software. Ramachandran plot analysis revealed that 99.7% of residues were in the allowed regions. High overall quality factor scores of ERRAT (93.41%) and Prosa Web results which confirmed the model to fall within the range of experimentally determined structure verified the stereochemical quality and reliability of the developed 3D model of vaccine construct (Figure 5).

### 3.10. Disulphide Bridging for Vaccine Protein Stability

**Figure 6:**
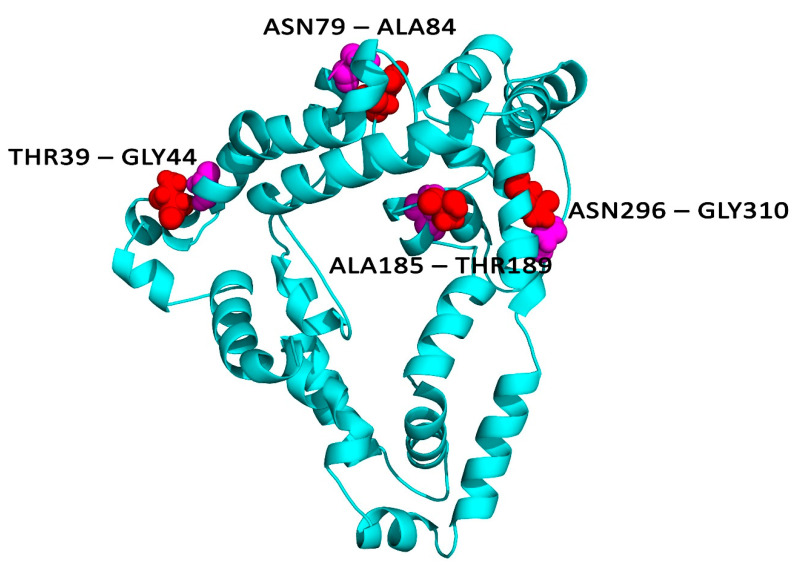
Disulphide engineering of the vaccine protein. Residue pairs showed in red (THR39, ASN79, ALA185, ASN296) and magenta (GLY44, ALA84, THR189, GLY310) spheres were mutated to Cysteine residues to form disulphide bridge between them.

A total of 55 pairs of residues were found that could be used for disulphide engineering which is given in Table S9. However, after evaluating other parameters such as energy and χ^3^, only a pair of residuals was finalized because their value falls below the allowable range, i.e., the value of energy should be less than 2.2 kcal/mol and χ^3^ angle should be in between −87 and +97 degree [39]. Therefore, a total of four mutations were generated on the residue pairs, THR39-GLY44, ASN79-ALA84, ALA185-THR189, and ASN296-GLY310 for which the χ^3^ angle and the energy were −86.78 degree and 1.91 kcal/mol, respectively, as mentioned in Figure 6.

### 3.11. Molecular Docking Analysis

**Figure 7:**
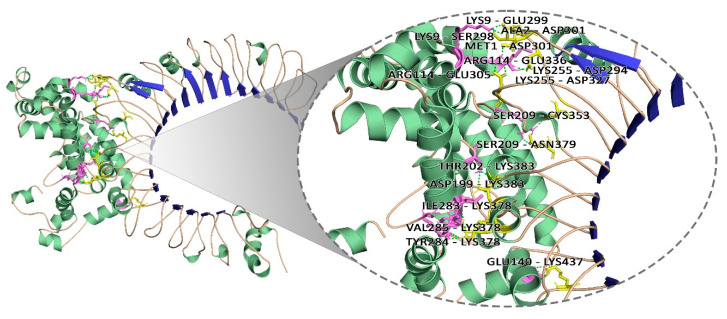
Molecular interaction of multi-epitope vaccine constructs with TLR2.

It was demonstrated that TLR-2 contributes to a protective innate immune response to *S. aureus* infection in several experiments. It is reported that lipoproteins play an important role in TLR2 activation by staphylococci. TLR2 is also a major player in staphylococcal disease through its diverse roles in various professional phagocyte functions: PMN stimulation and adhesion molecule expression, chemotaxis and chemoattractant receptor expression, and phagocytosis and ligand detection [45,46,47]. Therefore, the final vaccine construct was docked with TLR-2 (PDB Id: 3A7C) using the Cluspro server. The ribbon representation and surface view of the docked complex with the lowest energy score of −799.3 as viewed using Pymol is shown in Figure 7. The center energy between the ligand and receptor was −756.6. High binding affinity was observed between the vaccine construct and the immune receptor as displayed by multiple strong hydrogen bond (H-bond) interactions with a distance of 1.7Å to 2.5Å range between the interacting residues. The residues of docked vaccine-TLR2 complex showing H-bond interactions were LYS9—GLU299, LYS9—SER298, ALA2—ASP301, MET1—ASP301, ARG114—GLU305, ARG114—GLU336, LYS255—ASP294, LYS255—ASP327, SER209—CYS353, SER209—ASN379, THR202—LYS383, ASP199—LYS383, ILE283—LYS378, VAL285—LYS378, TYR284—LYS378, GLU140—LYS437 with a distance of 1.8Å, 2.4Å, 2.1Å, 2.1Å, 2.1Å, 2.0Å, 1.8Å, 2.5Å, 2.6Å, 2.2Å, 1.7Å, 1.8Å, 1.7Å, 2.3Å, 1.8Å, and 1.8Å, respectively.

### 3.12. MD Simulation

**Figure 8:**
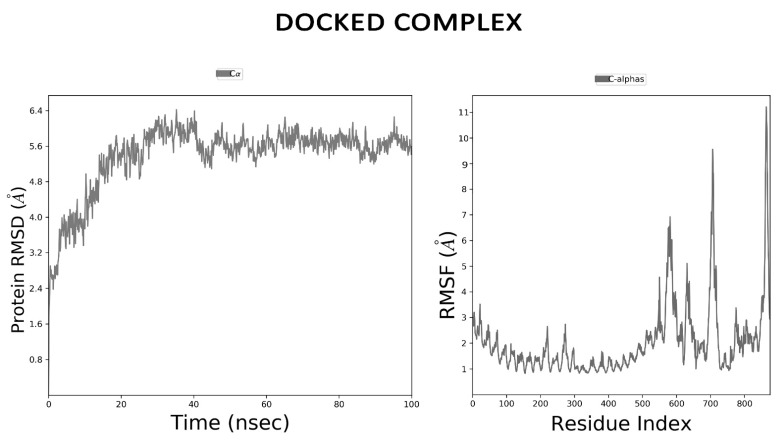
Root mean square deviation (RMSD) and root mean square fluctuation (RMSF) analysis of protein backbone and side chain residues of MD simulated vaccine construct.

The stability of the docked complex was determined by performing molecular dynamics simulation for100 ns using the Desmond tool from the Schrodinger package. MD was performed in the NPT ensemble at the temperature of 300 K and 1.01325 bar pressure over 100 ns with recording intervals of 1.2 ps for energy and 20 ps for trajectory. All other parameters were set at default. The root mean square deviation (RMSD) and root mean square fluctuation (RMSF) of the complex was calculated by trajectory analysis using the simulation interaction diagram. The stable RMSD (Figure 8) after 40 ns affirmed the protein-receptor complex attained equilibrium and tend to display a stable trend thereafter. While the RMSF plot of the vaccine showed more fluctuations than the receptor TLR-2 (residues ranging from 0–575 in the RMSF plot of Figure 8) indicating that the structure of the vaccine made significant movements to refine the interaction with the receptor to elicit a higher immune response. The RMSF plot also confirmed the stability of the structure.

### 3.13. Immune Simulation of the Vaccine Construct

**Figure 9:**
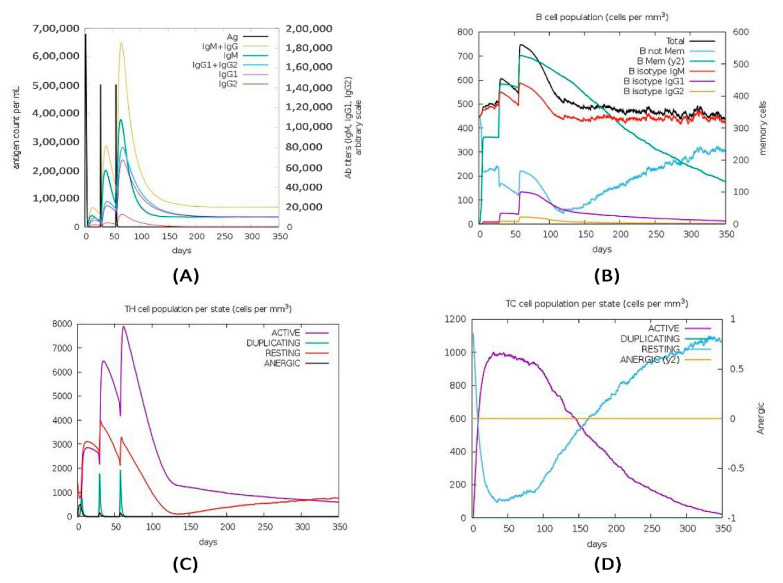
The immune simulation results of the designed vaccine construct using a C—immsim server: (**A**) Depicts increase in Immunoglobulins production in response to exposure to antigen injections with a marked decrease in the antigen concentration; (**B**) Showed the B—cell populations with a marked increase in the memory and non-memory immunoglobulins; (**C**,**D**) Indicates increased levels in the populations of the active T-helper and T-cytotoxic cells per state after the injections, respectively. The resting state indicates cells not exposed to antigen while the anergic state indicates tolerance of the T- cells to the antigen exposures.

C-ImmSim server was used to mimic the actual immune responses in the body upon exposure to the designed vaccine construct. Usually, the primary immune response arises as a result of the first contact with an antigen and the first antibody produced is mainly IgM, although a small amount of IgG is also produced. As shown in Figure 9 the amount of the IgM significantly increased during the first injection of the vaccine construct (antigen) as a primary immune response. The secondary immune response occurs as a result of the second and subsequent exposure to the same antigen and is characterized by increased levels of IgM and IgG. Furthermore, there was a noticeable increase in the level of IgM+IgG and decreased level of the antigen. Moreover, there were striking increases in the level of IgM, IgG1+IgG2, and IgG1 (Figure 9). These findings confirmed that the antibodies had a greater affinity to the vaccine construct (antigen) and would develop strong immune memory. Consequently, this resulted in increased clearance of the antigen upon subsequent exposures. Regarding the cytotoxic and helper T lymphocytes, high response in the cells populations with corresponding memory development was witnessed. Most importantly the population of the Helper T lymphocytes remained higher during all exposure time. In the IFN- γ induced epitopes prediction, the results showed a high IFN- γ concentration score compared to the other cytokines. The Simpson index D demonstrated the level of danger when the cytokines level increased, which may result in complications during the immune response.

### 3.14. In Silico Cloning and Optimisation

**Figure 10:**
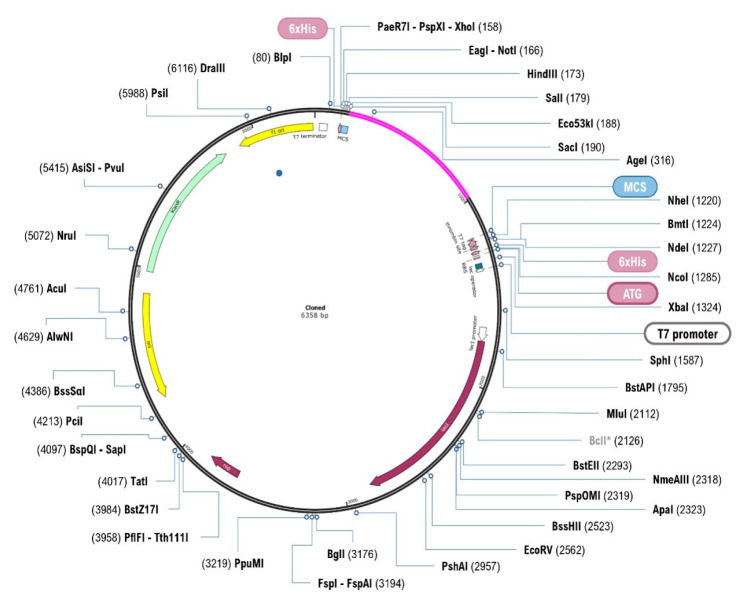
Restriction cloning of final multi-epitope vaccine 651 by using pET28a (+) expression vector in the in silico space. Black circle indicates the vector, and the magenta part is the place where the vaccine is inserted.

The subunit vaccine protein was the first codon optimized in order to clone the intended vaccine construct into an expression vector using an in silico technique. The optimized codon sequence has a length of 981 nucleotides. The optimized gene had a CAI value of 0.98, which was deemed ideal for expression in the target expression organism. Furthermore, the gene average GC content is 48.52 percent, which is within the optimum percentage range (30–70 percent). In addition, as illustrated in Figure 10, the optimized codon was inserted between the *EcoR*I (158) and *BamH*I (988) restriction sites of the *E. coli* vector pET28a (+). As a result, the final length of the clone was 6358 bp in total.

## 4. Discussion

The *S. aureus* bacteria can cause a variety of infections in humans, specifically the incidence of nosocomial infections caused by *S. aureus* has increased globally over the last two decades [48]. Making a vaccine that can prevent *S. aureus* infection has proven to be challenging. *S. aureus* is a nosocomial organism that has adapted an array of armaments specifically focused on subverting the human immune system. No single virulence factor is required for an infection that can be targeted as a vaccine is known. Variability in expression levels, resulting in a highly fluctuating surfacome, provides an easy way for bacteria to avoid immune effectors. *S. aureus* is notorious for causing spontaneous reinfection with the same strain, indicating natural infection does not readily induce acquired immunity. In spite of this, the scientific community has continued to innovate and develop diverse and complex vaccine designs in order to evoke various arms of the immune system and to tackle many *S. aureus* virulence mechanisms. There are many *S. aureus* vaccines currently engaged in various stages of clinical trials. Pier et al. have clearly mentioned the *S. aureus* vaccines. *S. aureus* strains can express either one of two capsular polysaccharides, CP5 or CP8, or neither, along with the poly-N-acetyl glucosamine (PNAG) surface polysaccharide antigen. PNAG was extensively evaluated in pre-clinical settings. Overall, substantial evidence for both protective immunity and function as a virulence factor in experimental *S. aureu*s infection scenarios was established, which, although hopeful, nonetheless represents findings equivalent to those for previous *S. aureus* vaccines that failed in human trials. Vaccines against CP antigens, immunity to ClfA, and vaccination against IsdB have all shown protection from lethality in mice but these vaccines have all failed in human trials [16]. Currently, Novadigm’s NDV3-A vaccine is in a phase II trial that consists of the N-terminal part of the *Candida albicans* cell-wall protein Als3p adjuvanted with alum provides cross-protection against *S. aureus* during murine models of bacteremia due to cross-kingdom antigen overlap [49,50,51]. A prior phase I trial by GSK investigating the safety and immunogenicity of a four-component vaccine containing: CP5, CP8, Hla, and ClfA was found to have no effect on rates of *S. aureus* carriage over two years [52] and also induced strong IgG responses in recipients. Olymvax has developed an *S. aureus* vaccine named rFSAV currently in phase II trials (CTR20181788) which is composed of five recombinant *S. aureus* antigens: Hla, SEB, MntC, IsdB, and SpA and showed promising efficacy in preclinical murine experiments. Integrated Bio Therapeutics has developed a heptavalent *S. aureus* vaccine consisting of seven *S. aureus* toxoids: Hla, Panton–Valentine Leukocidin (PVL) F and S subunits, Leukocidin A/B, SEA, SEB, and Toxic shock syndrome toxin 1. Preclinical data have shown that this vaccine, named IBT-V02, confers protection to both mice and rabbits against *S. aureus* skin infection, with protection being entirely mediated by vaccine-induced antibodies [53].

In addition, *Staphylococcus aureus* has long been associated with livestock. Livestock can be carriers of *S. aureus* and can also become infected. The best-known infection is bovine mastitis. The discovery of the methicillin-resistant *S. aureus* belonging to sequence type (ST) 398 boosted interest in livestock-associated *S. aureus*. However, there is also an exchange of strains between the reservoirs. Livestock-associated and human-associated strains share virulence factors but have also distinct virulence factors that appear to be important in host adaptation. The majority of these virulence factors were identified in isolates of human origin, and only a few studies have investigated virulence genes in non-ST398 *S. aureus* from chickens and cows. However, some novel virulence factors have recently been identified in mastitis and livestock-associated *S. aureus*. Ikawaty et al. investigated the presence of the SdrE protein in *S. aureus*-infected cows [54,55].

Further, the number of staphylococcal strains carrying multiple antibiotic resistances is gradually increasing across the world, and no preventive licensed vaccine has been approved yet. Therefore, there is an urgent need to develop vaccines that can efficiently promote protective immunity to target the pathogen for future therapies. Immunoinformatics is considered as a promising, faster, and reliable approach to screen cocktails of T-cell and B-cell epitopes that are more effective than single antigen or traditional deactivated pathogens vaccines and also flee the responses against undesirable epitopes in the antigen [56,57].

Hence, in the present study, we used to have a plethora of computational tools to identify epitopes from cell wall adherence proteins, SdrD, and SdrE of *S. aureus*. It is reported that the target proteins are effective vaccine candidates against several *S. aureus* strains as they play a vital role in promoting cell adhesion, acquisition of nutrients, and evasion of host immune responses. The epitopes finally identified for inclusion in the vaccine construct were observed to exhibit antigenicity, allergenicity, toxicity, and immunogenicity. To improve stability and immunogenicity, the vaccine epitopes were linked together using specific linkers. Linkers play an important role in the functional and structural behavior of fusion proteins, as well as in the representation of specific epitopes in the vaccine overall structure [58]. Subunit vaccines lack immunogenicity due to the small number of epitopes involved, but this can be improved with the use of an adjuvant [59]. To enhance the immunogenicity, Phenol soluble modulin α4 (Accession no. A9JX08) protein, a TLR 2 agonist was used as an adjuvant. Phenol-soluble modulins (PSMs) are a family of cytolytic peptide toxins which have multiple roles in staphylococcal virulence ranging from mediating phagosomal escape of phagocytes to deletion of the psm α operon for pathogenesis [15,60]. Hence, they are suggested to be ideal adjuvants that can maximise vaccine immunogenicity without compromising tolerability or safety. Furthermore, in a recent Immunoinformatics study for designing a novel multi-epitope vaccine against Staphylococcus aureus, phenol-soluble modulin alpha 4 was applied as the adjuvant [61]. Following the same line, in this study in an endeavor to develop a novel vaccine construct against *S. aureus*, phenol-soluble modulin alpha 4 was employed as an adjuvant. Later the vaccine construct was tested for antigenicity and allergenicity and was shown to be antigenic and non-allergic since vaccines with multiple epitopes are often poorly immunogenic and require coupling to adjuvant.

The final vaccine construct containing a cocktail of 2 B-Cell, 2 HTL and 20 T-Cell epitopes along with appropriate linkers and adjuvant possessed 327 residues and a molecular weight of 35.60 kDa. The theoretical PI was 5.79, indicating that the vaccine protein is very acidic in nature. The vaccine protein’s instability index was 24.73, which was well within the range of a stable protein. The instability index is a measure of the stability of an ideal vaccine, with a value less than 40 indicating that it is stable and a value greater than 40 indicating that it is unstable [62]. The total numbers of negatively and positively charged residues were 36 and 35, respectively. Additionally, the multi-epitope vaccine provided solubility indexes greater than the average probabilities of the SOLpro server indicating the solubility of the vaccine construct. Interferon-γ epitopes were even identified in the vaccine which confirmed the potential of vaccine to initiate Interferon-γ exhibiting both immunomodulatory and immunostimulatory actions. Additionally, the vaccine construct provided greater population coverage, covering approximately 98.95 percent of the world population.

The three-dimensional structure of the vaccine construct was determined using homology modelling followed by molecular docking and MD simulation using Desmond package of Schrodinger Maestro software for 100 ns to examine the stability of the interactions with its specific TLR2 immune receptor. One of the best characterized TLRs is TLR2 which initiates responses against a varied range of ligands and also are able to activate innate immune response by inducing the synthesis of pro-inflammatory mediators, in response to challenge with *S. aureus* [63].

The resulting RMSD and RMSF plots indicated the strong binding affinity of vaccine construct with receptor and with the minimum deviation Disulfide engineering is important for protein folding and stability. Additionally, structural disulfide engineering decreases the possible number of conformations for a given protein, resulting in decreased entropy and increased thermostability. In the developed vaccine construct, a total of four mutations were generated on the residue pairs, THR39-GLY44, ASN79-ALA84, ALA185-THR189, and ASN296-GLY310 for which the χ^3^ angle and the energy were −86.78 degree and 1.91 kcal/mol, respectively. We observed that disulphide engineering made the vaccine protein more stereochemically stable and also improved the interaction of the peptides with the TLR receptor as evident by docking and molecular dynamics simulation study. These observations are in concordance with several immunoinformatics studies where disulphide engineering have increased the affinity of vaccine peptides with receptor molecule [64,65].

Furthermore, immune simulation using the C-ImmSim server was performed to simulate the typical immune responses. Largely, there was a marked increase in the immunoglobulins coincided with a frequent injection of the vaccine construct. This result indicated the development of memory B-cells. Additionally, the level of the active T cytotoxic and T-helper lymphocytes was observed to escalate significantly supporting the enhancement of humoral and adaptive immune responses. The level of the IFN-γ was also witnessed to remain high at peak level during the injection times.

Lastly, the expression of the vaccine construct in a suitable *E. coli* expression vector is pivotal for the production of recombinant proteins [66,67]. The designed vaccine construct was reverse transcribed and adapted for *E. coli* strain K12 before cloning into the pET28a (+) vector. The codon adaptability index (0.98) and the GC content (48.52%) revealed high-level expression of the protein in bacteria. The vaccine construct gene was typically cloned in the vector in the multiple cloning sites. This finding confirmed the successful cloning of the vaccine protein.

## 5. Conclusions

In the present study, immunoinformatics approaches were used to develop an effective vaccine construct against *S. aureus* infections. With high-cost demands and numerous limitations for developing live, attenuated, or inactivated vaccine preparations for infectious agents such as *S. aureus*, these peptide-based vaccine candidates could be a relatively inexpensive and effective alternative option to fight Staphylococcal infection. The potential B- and T-cell epitopes were mapped from antigens of serine–aspartate repeat-containing cell wall adherence proteins, SdrD and SdrE. The developed multiepitopic vaccine with a good immune response and wide population coverage and exhibiting high antigenicity, non-toxicity, and non-allergenicity could be a potential candidate for clinical trials. In silico immune simulation showed an immune response in accordance with the clearing of antigen. Computational cloning in PET28a (+) plasmid demonstrated good protein expression. The obtained results indicated that the proposed vaccine holds a high potential to elicit humoral and cellular immunity and is a promising candidate for further in vitro and in vivo studies.

## Figures and Tables

**Table 1:** Predicted B-cell epitopes from *S. aureus* Serine–aspartate repeat-containing cell wall adherence proteins, SdrD and SdrE and their corresponding immunogenic properties.

Uniprot_ID	B-Cell Epitope	Position	Score	Antigenicity Score	Toxicity	Hydrophobicity	Hydropathicity	Hydrophilicity	Charge	Mol wt.
O86488	NEENKKVDAK	173	0.58	2.4006	Non-toxin	−0.57	−2.32	1.64	0	1174.41
O86489	DTGGGDGTVK	593	0.58	4.1781	Non-toxin	−0.17	−0.97	0.67	−1	906.06

**Table 2:** Predicted CTL epitopes from *S. aureus* cell wall adherence proteins, SdrD and SdrE to design multi-epitope vaccine construct with their corresponding MHC Class I alleles and their immunogenic properties.

Uniprot_ID	CTL Epitope	Alleles	Position	Score	Antigencity Score	Immunogenicity	Toxicity
O86488	SGGAGQEVY	HLA-A*01:01	562	1.841	2.6468	0.08295	Non-toxin
	TLKNENGEV	HLA-A*02:01	822	4.4391	1.4625	0.102	Non-toxin
	VTSGNDTEK	HLA-A*03:01	977	1.3119	2.012	0.08624	Non-toxin
	DYRVEFSNL	HLA-A*24:02	629	0.9136	1.2237	0.12264	Non-toxin
	NTRMRIAAV	HLA-A*26:01	229	1.307	0.7456	0.07166	Non-toxin
	APKRLNTRM	HLA-B*07:02	224	0.0577	1.3117	0.02819	Non-toxin
	DAKTESTTL	HLA-B*08:01	180	0.1135	1.9205	0.03373	Non-toxin
	SKNDVEFNV	HLA-B*27:05	370	4.146	1.2062	0.24969	Non-toxin
	SQITVDDKV	HLA-B*40:01	279	1.829	0.8785	0.03606	Non-toxin
	SGDYFTIKY	HLA-B*58:01	289	3.063	1.6238	0.14034	Non-toxin
O86489	KGDTMTINY	HLA-A*01:01	323	0.311	1.8025	0.02034	Non-toxin
	KTATEDTSV	HLA-A*02:01	142	3.039	1.4951	0.10624	Non-toxin
	NTNNDVTTK	HLA-A*03:01	160	1.096	2.0046	0.10729	Non-toxin
	KYSLGDYVW	HLA-A*24:02	830	0.105	0.4525	0.01002	Non-toxin
	TSQNVTVDY	HLA-A*26:01	415	1.098	1.3552	0.08043	Non-toxin
	TPAGYTPTV	HLA-B*07:02	786	0.194	0.9088	0.09306	Non-toxin
	EQQIYVNPL	HLA-B*08:01	449	1.332	0.7245	0.11964	Non-toxin
	KQTIEQQIY	HLA-B*27:05	445	4.415	0.8527	0.11498	Non-toxin
	DKQTIEQQI	HLA-B*39:01	444	1.591	1.2024	0.05987	Non-toxin
	IEQQIYVNP	HLA-B*40:01	448	2.472	0.7436	0.00302	Non-toxin

**Table 3:** Predicted HTL epitopes from *S. aureus* cell wall adherence proteins, SdrD and SdrE to design a multi-epitope vaccine construct with their corresponding MHC Class II alleles and their immunogenic properties.

Uniprot_ID	MHC II Epitope	Alleles	Position	IC50 Value	Percentile Rank	Antigenicity Score	Toxicity	Hydrophobicity	Hydropathicity	Hydrophilicity	Charge	Mol. wt.
O86488	KRLNTRMRIAAVQPS	HLA-DQA1*01:02, HLA-DQB1*06:02, HLA-DPB1*01:01, HLA-DRB1*01:01, HLA-DRB1*09:01,HLA-DRB3*02:02, HLA-DRB1*13:02, HLA-DRB1*11:01, HLA-DRB1*04:01, HLA-DRB1*12:01, HLA-DPA1*03:01, HLA-DPB1*04:02, HLA-DRB1*04:05, HLA-DRB1*15:01, HLA-DQA1*01:01, HLA-DQB1*05:01, HLA-DRB1*08:02, HLA-DPA1*02:01, HLA-DPB1*14:01, HLA-DPA1*01:03, HLA-DPB1*04:01, HLA-DQA1*05:01, HLA-DQB1*03:01, HLA-DQA1*04:01, HLA-DQB1*04:02, HLA-DPA1*02:01, HLA-DPA1*02:01, HLA-DPB1*05:01, HLA-DPA1*01:03, HLA-DPB1*02:01, HLA-DQA1*05:01, HLA-DQB1*02:01, HLA-DQA1*03:01, HLA-DQB1*03:02, HLA-DRB3*01:01, HLA-DRB5*01:01, HLA-DRB1*07:01, HLA-DRB4*01:01, HLA-DRB1*03:01	226–240	30	0.11	0.9801	Non-toxin	−0.38	−0.63	0.33	4	1741.3
O86489	DTEFTIDNKVKKGDT	HLA-DRB1*03:01, HLA-DRB3*01:01, HLA-DPA1*03:01, HLA-DPB1*04:02, HLA-DPA1*01:03, HLA-DPB1*02:01,HLA-DRB1*01:01, HLA-DRB1*09:01,HLA-DRB3*02:02, HLA-DRB1*13:02, HLA-DRB1*11:01, HLA-DRB1*04:01, HLA-DRB1*12:01,HLA-DRB1*04:05, HLA-DRB1*15:01, HLA-DQA1*01:01, HLA-DQB1*05:01, HLA-DRB1*08:02, HLA-DPA1*02:01, HLA-DPB1*14:01, HLA-DPA1*01:03, HLA-DPB1*04:01, HLA-DQA1*05:01, HLA-DQB1*03:01, HLA-DQA1*04:01, HLA-DQB1*04:02, HLA-DPA1*02:01, HLA-DPB1*01:01, HLA-DPA1*02:01, HLA-DPB1*05:01,HLA-DQA1*05:01, HLA-DQB1*02:01, HLA-DQA1*03:01, HLA-DQB1*03:02, HLA-DQA1*01:02, HLA-DQB1*06:02, HLA-DRB5*01:01, HLA-DRB1*07:01, HLA-DRB4*01:01,	312–326	88	0.88	1.2192	Non-toxin	−0.35	−1.35	0.95	−1	1711.07

## Data Availability

Data is contained within the article or Supplementary Material.

## Supplementary Materials

The following are available online at https://www.mdpi.com/article/10.3390/vaccines9091038/s1, Table S1: The protein sequence of the potential vaccine target against *S. aureus*, Table S2: Predicted B-cell epitopes from *S. aureus* Serine–aspartate repeat-containing proteins and their corresponding immunogenic properties. The final selected epitopes to design multi-epitope vaccine construct from proteins were highlighted in green colour, Table S3: Predicted CTL epitopes from *S. aureus* cell wall adherence proteins, SdrD and SdrE with their corresponding MHC Class I alleles and their immunogenic properties. The final selected epitopes to design multi-epitope vaccine construct from proteins were highlighted in green colour, Table S4: Predicted HTL epitopes from *S. aureus* cell wall adherence proteins, SdrD and SdrE with their corresponding MHC Class II alleles and their immunogenic properties. The final selected epitopes to design multi-epitope vaccine construct from proteins were highlighted in green colour, Table S5: Final epitopes of the vaccine construct with their basic properties., Table S6: Population Coverage analysis of the final vaccine construct using the population coverage analysis tool of the IEDB database by keeping the default parameters on (109 countries covering 16 different geographical regions), Table S7: Physicochemical Properties of final vaccine construct as predicted by ProtParam tool, Table S8: The predicted IFN-γ inducing epitopes from the proposed vaccine. The amino acid residues of the vaccine, Table S9: Disulfide engineering of the final multiepitope vaccine.
