# Supplementary material for: Development of a Conserved Chimeric Vaccine for Induction of Strong Immune Response against Staphylococcus aureus Using Immunoinformatics Approaches
Source: Vaccines (Basel). 2021 Sep 18;9(9):1038. doi: 10.3390/vaccines9091038 (PMC8470666; doi:10.3390/vaccines9091038)
Supplement: Supplementary file 1 [file vaccines-09-01038-s001.zip › Table S1.docx]

Table S1: The protein sequence of the potential vaccine target against *S. aureus*.

>sp|O86488|SDRD_STAAE Serine-aspartate repeat-containing protein D OS=Staphylococcus aureus (strain Newman) OX=426430 GN=sdrD PE=1 SV=1

MLNRENKTAITRKGMVSNRLNKFSIRKYTVGTASILVGTTLIFGLGNQEAKAAESTNKEL

NEATTSASDNQSSDKVDMQQLNQEDNTKNDNQKEMVSSQGNETTSNGNKLIEKESVQSTT

GNKVEVSTAKSDEQASPKSTNEDLNTKQTISNQEALQPDLQENKSVVNVQPTNEENKKVD

AKTESTTLNVKSDAIKSNDETLVDNNSNSNNENNADIILPKSTAPKRLNTRMRIAAVQPS

STEAKNVNDLITSNTTLTVVDADKNNKIVPAQDYLSLKSQITVDDKVKSGDYFTIKYSDT

VQVYGLNPEDIKNIGDIKDPNNGETIATAKHDTANNLITYTFTDYVDRFNSVQMGINYSI

YMDADTIPVSKNDVEFNVTIGNTTTKTTANIQYPDYVVNEKNSIGSAFTETVSHVGNKEN

PGYYKQTIYVNPSENSLTNAKLKVQAYHSSYPNNIGQINKDVTDIKIYQVPKGYTLNKGY

DVNTKELTDVTNQYLQKITYGDNNSAVIDFGNADSAYVVMVNTKFQYTNSESPTLVQMAT

LSSTGNKSVSTGNALGFTNNQSGGAGQEVYKIGNYVWEDTNKNGVQELGEKGVGNVTVTV

FDNNTNTKVGEAVTKEDGSYLIPNLPNGDYRVEFSNLPKGYEVTPSKQGNNEELDSNGLS

SVITVNGKDNLSADLGIYKPKYNLGDYVWEDTNKNGIQDQDEKGISGVTVTLKDENGNVL

KTVTTDADGKYKFTDLDNGNYKVEFTTPEGYTPTTVTSGSDIEKDSNGLTTTGVINGADN

MTLDSGFYKTPKYNLGNYVWEDTNKDGKQDSTEKGISGVTVTLKNENGEVLQTTKTDKDG

KYQFTGLENGTYKVEFETPSGYTPTQVGSGTDEGIDSNGTSTTGVIKDKDNDTIDSGFYK

PTYNLGDYVWEDTNKNGVQDKDEKGISGVTVTLKDENDKVLKTVTTDENGKYQFTDLNNG

TYKVEFETPSGYTPTSVTSGNDTEKDSNGLTTTGVIKDADNMTLDSGFYKTPKYSLGDYV

WYDSNKDGKQDSTEKGIKDVKVTLLNEKGEVIGTTKTDENGKYCFDNLDSGKYKVIFEKP

AGLTQTGTNTTEDDKDADGGEVDVTITDHDDFTLDNGYYEEETSDSDSDSDSDSDSDRDS

DSDSDSDSDSDSDSDSDSDSDSDSDSDRDSDSDSDSDSDSDSDSDSDSDSDSDSDSDSDS

DSDSDSDSDSDSDSDSDSDSDSDSDSDSDSDSDSDSDSDSDSDSDSDSDSDSDSDAGKHT

PVKPMSTTKDHHNKAKALPETGNENSGSNNATLFGGLFAALGSLLLFGRRKKQNK

>sp|O86489|SDRE_STAAE Serine-aspartate repeat-containing protein E OS=Staphylococcus aureus (strain Newman) OX=426430 GN=sdrE PE=1 SV=1

MINRDNKKAITKKGMISNRLNKFSIRKYTVGTASILVGTTLIFGLGNQEAKAAENTSTEN

AKQDDATTSDNKEVVSETENNSTTENNSTNPIKKETNTDSQPEAKKESTSSSTQKQQNNV

TATTETKPQNIEKENVKPSTDKTATEDTSVILEEKKAPNNTNNDVTTKPSTSEPSTSEIQ

TKPTTPQESTNIENSQPQPTPSKVDNQVTDATNPKEPVNVSKEELKNNPEKLKELVRNDS

NTDHSTKPVATAPTSVAPKRVNAKMRFAVAQPAAVASNNVNDLIKVTKQTIKVGDGKDNV

AAAHDGKDIEYDTEFTIDNKVKKGDTMTINYDKNVIPSDLTDKNDPIDITDPSGEVIAKG

TFDKATKQITYTFTDYVDKYEDIKSRLTLYSYIDKKTVPNETSLNLTFATAGKETSQNVT

VDYQDPMVHGDSNIQSIFTKLDEDKQTIEQQIYVNPLKKSATNTKVDIAGSQVDDYGNIK

LGNGSTIIDQNTEIKVYKVNSDQQLPQSNRIYDFSQYEDVTSQFDNKKSFSNNVATLDFG

DINSAYIIKVVSKYTPTSDGELDIAQGTSMRTTDKYGYYNYAGYSNFIVTSNDTGGGDGT

VKPEEKLYKIGDYVWEDVDKDGVQGTDSKEKPMANVLVTLTYPDGTTKSVRTDANGHYEF

GGLKDGETYTVKFETPTGYLPTKVNGTTDGEKDSNGSSVTVKINGKDDMSLDTGFYKEPK

YNLGDYVWEDTNKDGIQDANEPGIKDVKVTLKDSTGKVIGTTTTDASGKYKFTDLDNGNY

TVEFETPAGYTPTVKNTTADDKDSNGLTTTGVIKDADNMTLDRGFYKTPKYSLGDYVWYD

SNKDGKQDSTEKGIKDVTVTLQNEKGEVIGTTKTDENGKYRFDNLDSGKYKVIFEKPAGL

TQTVTNTTEDDKDADGGEVDVTITDHDDFTLDNGYFEEDTSDSDSDSDSDSDSDSDSDSD

SDSDSDSDSDSDSDSDSDSDSDSDSDSDSDSDSDSDSDSDSDSDSDSDSDSDSDSDSDSD

SDSDSDSDSDSDSDSDSDSDSDSDSDSDSDSDSDSDSDSDSDSDSDSDSDSDSDSDSDSD

SDSDSDSDSDSDSDSDSDSDSDSDSDAGKHTPVKPMSTTKDHHNKAKALPETGSENNGSN

NATLFGGLFAALGSLLLFGRRKKQNK
