# Supplementary material for: Development of a Conserved Chimeric Vaccine for Induction of Strong Immune Response against Staphylococcus aureus Using Immunoinformatics Approaches
Source: Vaccines (Basel). 2021 Sep 18;9(9):1038. doi: 10.3390/vaccines9091038 (PMC8470666; doi:10.3390/vaccines9091038)
Supplement: Supplementary file 1 [file vaccines-09-01038-s001.zip › Table S7.docx]

**Table S7.** Physicochemical Properties of final vaccine construct as predicted by ProtParam tool.

| **Parameters** | **Vaccine Construction Sequence** |
| --- | --- |
| Number of amino acids | 327 |
| Molecular weight | 35549.60 KD |
| Theoretical pI | 5.79 |
| Total number of negatively charged residues (Asp + Glu) | 36 |
| Total number of positively charged residues (Arg + Lys) | 35 |
| Instability index | 24.73 (Stable) |
| Aliphatic index | 70.03 |
| Grand average of hydropathicity (GRAVY) | -0.438 |
| The estimated half-life | 30 hours (mammalian reticulocytes, in vitro).  >20 hours (yeast, in vivo)  >10 hours (Escherichia coli, in vivo). |
| Amino acid composition | \| Ala (A) \| 56 \| 17.10% \| \| --- \| --- \| --- \| \| Arg (R) \| 8 \| 2.40% \| \| Asn (N) \| 19 \| 5.80% \| \| Asp (D) \| 20 \| 6.10% \| \| Cys (C) \| 0 \| 0.00% \| \| Gln (Q) \| 14 \| 4.30% \| \| Glu (E) \| 16 \| 4.90% \| \| Gly (G) \| 21 \| 6.40% \| \| His (H) \| 0 \| 0.00% \| \| Ile (I) \| 21 \| 6.40% \| \| Leu (L) \| 7 \| 2.10% \| \| Lys (K) \| 27 \| 8.30% \| \| Met (M) \| 5 \| 1.50% \| \| Phe (F) \| 5 \| 1.50% \| \| Pro (P) \| 10 \| 3.10% \| \| Ser (S) \| 11 \| 3.40% \| \| Thr (T) \| 32 \| 9.80% \| \| Trp (W) \| 1 \| 0.30% \| \| Tyr (Y) \| 32 \| 9.80% \| \| Val (V) \| 22 \| 6.70% \| \| Pyl (O) \| 0 \| 0.0% \| \| Sec (U) \| 0 \| 0.0% \| |
| Atomic composition | \| Carbon \| C \| 1584 \| \| --- \| --- \| --- \| \| Hydrogen \| H \| 2446 \| \| Nitrogen \| N \| 412 \| \| Oxygen \| O \| 508 \| \| Sulfur \| S \| 5 \| |
| Secondary structure analysis | Alpha helix (Hh) 26.60%  Extended strand (Ee) 34.86%  Random coil (Cc) 38.53% |
